# Determination of the absolute CH_4_ adsorption using simplified local density theory and comparison with the modified Langmuir adsorption model

**DOI:** 10.1039/c8ra08586b

**Published:** 2018-12-11

**Authors:** Yeyu Zhang, Shaonan Zhang, Zhicheng Wang, Hucheng Deng, Minghui Qi, Xianfeng Peng, Yueliang Liu

**Affiliations:** College of Energy Resources, Chengdu University of Technology Chengdu 610059 China denghucheng@cdut.edu.cn +86 13402851216; State Key Laboratory of Oil and Gas Reservoir Geology and Exploitation Chengdu 610059 China; College of Geoscience and Technology, Southwest Petroleum University Chengdu 610501 China; Sichuan Institute of Coal Field Geological Engineering Exploration and Designing Chengdu 610072 China; Shale Gas Evaluation and Exploitation Key Laboratory of Sichuan Province Chengdu 610051 China; School of Petroleum Engineering, China University of Petroleum (East China) Qingdao Shandong 266580 China sdliuyueliang@163.com

## Abstract

Accurately determining the adsorbed amount of CH_4_ on shale is significant for understanding the mechanisms of shale gas storage and shale methane recovery from shale gas reservoirs. Excess CH_4_ adsorption is measured using the thermogravimetric method. Simplified local density (SLD) theory is applied to calculate the adsorbed CH_4_ density to obtain the absolute adsorption. Moreover, the modified Langmuir adsorption model is employed to fit the excess adsorption to describe the absolute adsorption. The adsorbed CH_4_ density from the SLD model is affected by the system pressure and temperature, while such density obtained from the modified Langmuir model is only a function of temperature. Compared to the modified Langmuir model, the SLD model can better capture the adsorbed CH_4_ density, which allows accurate determination of the absolute CH_4_ adsorption.

## Abstract 2

Accurately determining the adsorbed amount of CH_4_ on shale is significant for understanding the mechanisms of shale gas storage and shale methane recovery from shale gas reservoirs.

## Graphical abstract

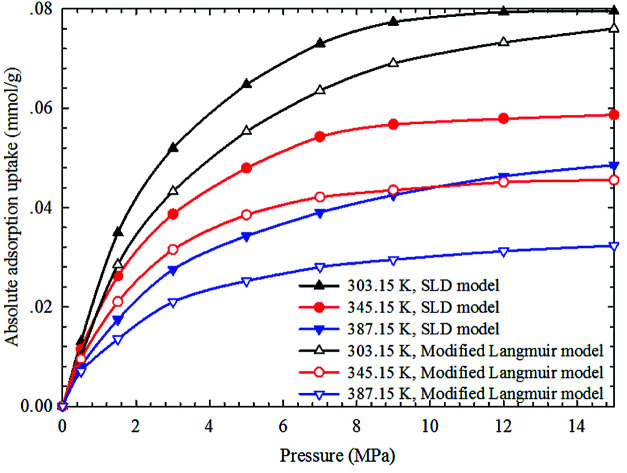

## 1. Introduction

Shale gas is one kind of unconventional energy resource, which has become an increasingly important energy in recent years. Shale reservoirs generally exhibit some typical characteristics of extremely low permeability, and heterogeneity.^1^ Shale generally contains a large amount of kerogen, which can result in the significant adsorption of shale gas on the organic shale surface.^2^ Accurately measuring the amount of adsorbed shale gas is quite important for the estimation of shale gas storage and the development of shale gas reservoirs.

As is known, CH_4_ is a common component existing in shale fluid. In shale gas reservoirs, CH_4_ is generally stored in three different states, which is clarified as free-gas state in nanopores, absorbed-gas state in kerogen, and adsorbed-gas state on pore surface.^3–5^ It has been found that the adsorbed CH_4_ can take 20–85 vol% accounting for the total gas amount.^4^ Recently, extensive studies are implemented to measure the CH_4_adsorption on shale samples. Volumetric method^6–10^ and thermogravimetric (TGA) method^3,11,12^ are two main approaches applied for measuring the CH_4_ adsorption isotherms on shale. TGA method enables to measure the weight difference as accurate as 1 μg. Thereby, compared to volumetric method, TGA method is more accurate in measuring the amount of adsorbed CH_4_ on shale samples.

However, the laboratory measurement only provides the excess adsorption. It has proposed that the measured excess adsorption has possible underestimation of the amount of adsorbed CH_4_.^12^ Generally, the measured excess adsorption is transformed to the absolute values, which reflects the actual adsorbed amount of CH_4_ on shale.^4,12^ The density of adsorbed CH_4_ is usually employed to make this conversion. Due to the difficulty in measuring such density directly, some constant values are generally used to represent the density of adsorbed CH_4_. For instance, the density of adsorbed CH_4_ is suggested to be the liquid CH_4_ density at the room boiling point, *i.e.*, 420 kg m^−3^.^13–17^ However, it is proved that the density of adsorbed CH_4_ is strongly affected by temperature, pressure, and pore size.^12,18,19^ Most recently, some correlation models, such as the modified Langmuir adsorption model, Dubinin–Radushkevitch equation, Ono–Kondo models, and Supercritical Dubinin–Radushkevitch equation, are widely employed to obtain the absolute adsorption by adjusting the adsorbed CH_4_ density.^19–22^ Due to the simplicity for usage, the modified Langmuir adsorption model is extensively used in obtaining the absolute adsorption.

Molecular simulation is also employed to investigate the density of adsorbed CH_4_ on shale. By specifically considering the fluid-pore wall interactions, molecular simulation provides the fundamental mechanisms of CH_4_ adsorption in organic pores. Recently, Liu *et al.* (2018)^12^ measured the excess adsorption of two hydrocarbon-species, *i.e.*, methane and *n*-butane, on two typical shale samples; the molecular simulation method was then used to calculate the adsorbed CH_4_ density. Such density is then applied to describe the absolute adsorption by correcting the measured excess. Base on their simulation results, they observed that the adsorbed CH_4_ density is affected by temperature, pressure, and pore size. Furthermore, Ambrose *et al.* (2012)^19^ also observed the adsorbed CH_4_ density changes with temperature, pressure, and pore size. Although molecular simulation could accurately determine the adsorbed phase density, the computation is quite expensive compared to the conventional methods. Simplified local density (SLD) theory specifically takes into consideration the fluid/pore surface interactions, which can thereby determine the density of adsorbed CH_4_ accurately. Compared to molecular simulation method, SLD model significantly reduces the computational time.

In this study, the excess CH_4_ adsorption is measured on the typical shale samples. The modified Langmuir adsorption model and the SLD model are then employed to capture the absolute adsorption based on the excess adsorption. As the previous study,^23^ the SLD model captures the absolute adsorption by obtaining the density of adsorbed CH_4_ on shale. The modified Langmuir adsorption model describes the absolute adsorption by accurately fitting the excess adsorption. The performance of the modified Langmuir adsorption model is then evaluated by comparing with the SLD model. The objectives of this study are: (1) to assess the validity of the widely used modified Langmuir adsorption model in determining the absolute CH_4_ adsorption on shale; (2) to propose a practical method, *i.e.*, the SLD model, to determine the absolute CH_4_ adsorption. In our SLD model, the carbon-slit pore model is employed to describe the organic pores.

## 2. Experimental section

### 2.1 Materials

The CH_4_ used in this work has a purity of 99.95 mol%. The two shale samples used are obtained from Longmaxi formation. Before experiment, the shale samples are sealed to avoid the moisture.

### 2.2 Characterization of the shale samples

#### (1) N_2_ adsorption/desorption tests

In this study, N_2_ adsorption/desorption tests are conducted to characterize pore size distribution of both shale samples. The Gas Adsorption Analyzer (Quantachrome, America) is employed for this characterization. By analyzing the adsorption data measured at 77.0 K, we can obtain the pore size distribution as well as the specific surface area.

#### (2) TOC measurement

To obtain the TOC content of both shale samples, an elemental analyzer is employed. The organic carbon in shale is first formed by CO_2_; a non-dispersive infrared analyzer is then applied to measure the total molar amount of CO_2_.

#### (3) Scanning election microscopy (SEM)

In this work, the Hitachi SEM setup is applied to obtain the surface morphology of both shale samples. Before the SEM scanning, argon ion is used to polish the shale surface. Then, the shale surface is then covered by a golden film to improve the conductivity. The shale samples are scanned at a voltage of 20.0 kV.

### 2.3 Measurement of the excess adsorption

In this adsorption experiment, we measure the excess CH_4_ adsorption with a thermalgravimetric (TGA) analyzer at the temperatures of 303.15, 345.15, and 387.15 K, and pressures as high as 15.0 MPa. With the TGA method, the measured excess adsorption uptake can be expressed as,^24^

1 *M*_ex_ = *M*_ad_ − *ρV*_ad_

where *M*_ex_ represents the excess adsorption uptake; *M*_ad_ represents the adsorbed uptake, which is recognized as the uptake of the absolute adsorption; *ρ* represents the CH_4_ density in bulk; and *V*_ad_ represents the adsorbed volume of CH_4_. We find that the measured excess adsorption is smaller than the adsorbed CH_4_ adsorption on shale.

The adsorbed volume of CH_4_ can be given by,2
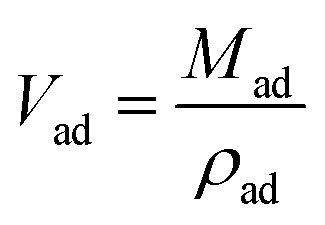


Substituting eqn (2) into (1), we can obtain the expression for absolute adsorption, which represents the actual adsorption uptake of CH_4_ on shale,3
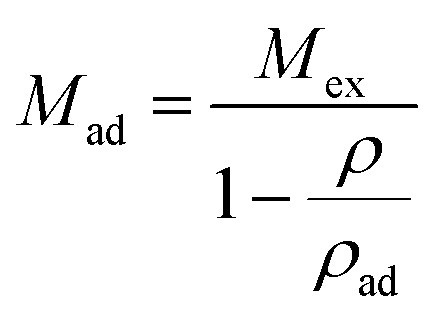


To confirm the reliability and reproductivity of the measured data, we repeat each test twice, and it is found that the maximum deviation is always smaller than ±3.76% between two measuring runs.

### 2.4 Modified Langmuir adsorption model

As shown in eqn (1), the adsorbed CH_4_ density is important to obtain the absolute adsorption. Recently, three categories of conversion methods have been proposed to represent this quantity. One approach is to predetermine the adsorbed CH_4_ density as a constant value, which generally ranges between 0.373 g cm^−3^ (ref. 19 and 25) and 0.423 g cm^−3^.^3,26,27^ Our previous study has proved that this method is unphysically reasonable considering that the adsorbed CH_4_ density is generally influenced by system pressure, temperature, and pore size.^12^ Another approach is to determine this value by fitting a modified equation to the measured excess isotherm by adjusting the adsorbed CH_4_ density.^26–28^ Due to its simplicity and low computational cost, the modified Langmuir adsorption model is widely used for fitting excess isotherms and then calculating the absolute isotherms.^26,27^ This model is based on the assumption that CH_4_ generally exhibits monolayer adsorption on carbon surface,^29,30^ which can be expressed as,^27^4
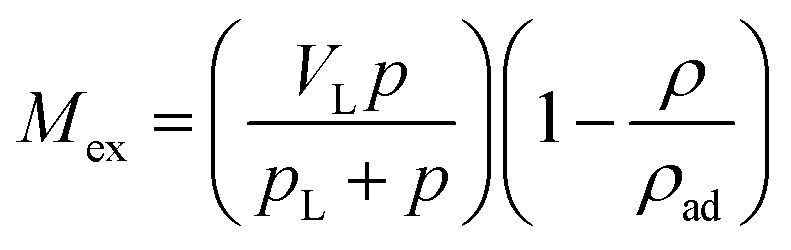
where *V*_L_ represents the maximum adsorbed amount of CH_4_; *p*_L_ represents the Langmuir pressure; *p* represents the system pressure. The first term 
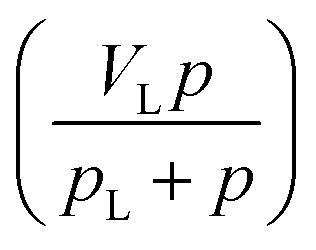
 in eqn (4) is the standard Langmuir equation. *ρ*_ad_ is initially determined by fitting eqn (4) to the directly measured excess adsorption. According to eqn (4), the absolute adsorption uptake is then calculated as,^27^5
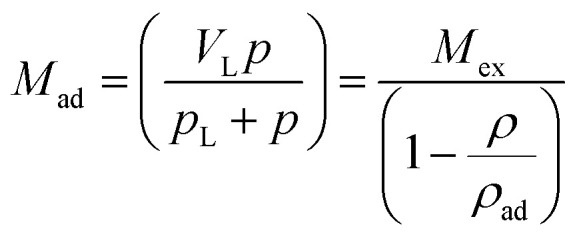


### 2.5 Simplified local density (SLD) model

The SLD model is originally proposed by Rangarajan *et al.* (1995),^31^ which is generally applied to describe gas adsorption on adsorbate surface over a wide pressure/temperature range. The SLD model specifically considers the fluid-pore surface and fluid–fluid interactions, which can accurately describe the gas adsorption on pore surface.^32^

The main assumptions proposed for the SLD model are summarized as below,

(1) Near the pore surface, the chemical potential at any point is equal to the chemical potential in bulk;

(2) At any point, the chemical potential at equilibrium is equal to the summation of potentials due to fluid-pore surface and fluid–fluid interactions;

(3) The fluid-pore surface potentials at any point do not correlate the total number of molecules around this point.

When adsorption reaches equilibrium, the gas chemical potential at position *z* is calculated as the summation of the potentials due to fluid-pore surface and fluid–fluid interactions; it is regarded as the bulk chemical potential.

6 *μ*(*z*) = *μ*_ff_(*z*) + *μ*_fs_(*z*) = *μ*_bulk_

where the subscript “ff” represents fluid–fluid interactions, “fs” represents fluid-pore surface interactions, and “bulk” represents the gas in bulk.

The bulk chemical potential of gas can be given as a function of fugacity by,7
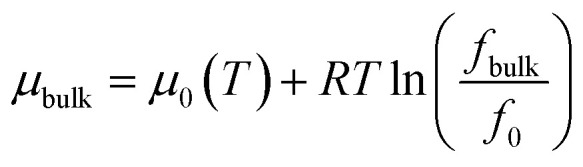
where *f*_bulk_ represents the fugacity of gas in bulk, *f*_0_ represents the fugacity at a reference state. The chemical potential due to fluid–fluid interaction is expressed as,8
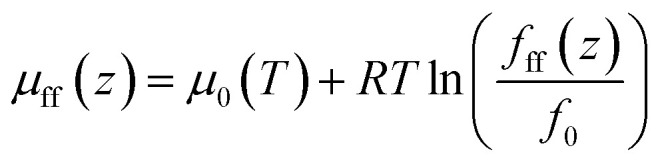
where *f*_ff_(*z*) represents the gas fugacity at position *z*; *f*_0_ represents the fugacity at the same reference state as that in eqn (7).

The gas chemical potential in nanopores due to the fluid-pore wall interaction is expressed as,^31^

9 *μ*_fs_(*z*) = *N*_A_[*Ψ*^fs^(*z*) + *Ψ*^fs^(*L* − *z*)]

where *Ψ*^fs^(*z*) and *Ψ*^fs^(*L* − *z*) represent the fluid-pore surface interactions from both walls of a pore; *L* represents the pore size; *N*_A_ represents Avogadro number.

The Lee's partially integrated 10–4 Lennard-Jones potential^33^ is applied to represent the fluid-pore surface interaction,10
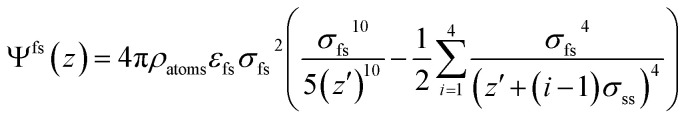
where *ρ*_atoms_ is the solid-atom density, 38.2 atoms per nm^2^;^34^*ε*_fs_ is the interaction parameter between fluid and pore surface; *σ*_fs_ is the fluid-pore surface molecular diameter, which can be calculated by *σ*_fs_ = (*σ*_ff_ + *σ*_ss_)/2, where *σ*_ff_ represent the molecular diameter of CH_4_, while *σ*_ss_ represents the carbon-interplanar distance. As for graphite, *σ*_ss_ is taken as0.355 nm; *z*' is the dummy coordinate, *z*' = *z* + *σ*_ss_/2.

Substituting eqn (8)–(10) into eqn (6), we can obtain the following,11
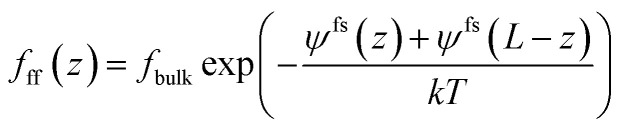
where *k* is the Boltzmann constant, 1.38 × 10^−23^ J K^−1^; and *T* is the absolute temperature.

The Peng–Robinson equation of state (PR-EOS) is employed to take into consideration the fluid–fluid interactions, which can be expressed as a function of density (*ρ*) as,12
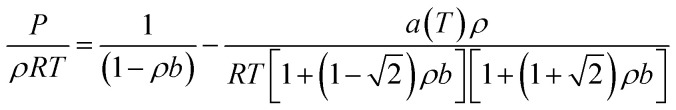
where13
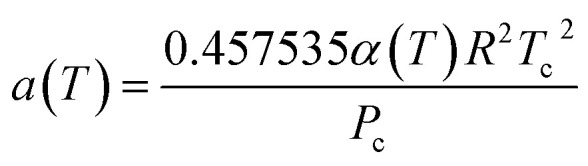
14
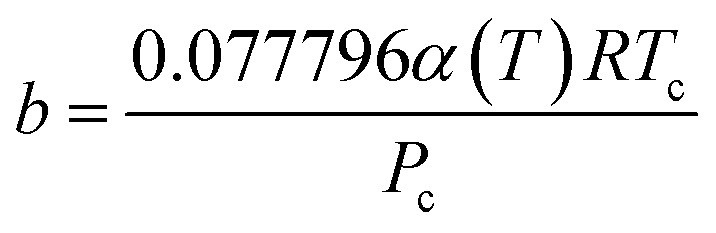


The term *α*(*T*) in eqn (13) can be expressed as.^35^15
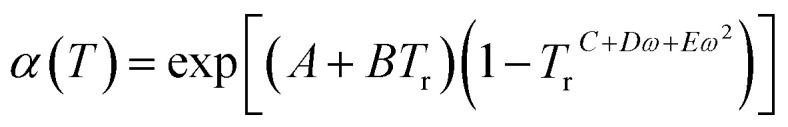
where *A*, *B*, *C*, and *D* represent the correlation parameters with the values fixed at 2.0, 0.8145, 0.508, and −0.0467, respectively. As for CH_4_, the value of the acentric factor (*ω*), the critical pressure (*P*_c_), the critical temperature (*T*_c_), and the molecular diameter are 0.0113, 4.6 MPa, 190.56 K, and 0.3758 nm, respectively.

Applying the PR-EOS, the gas fugacity in bulk is expressed as,16
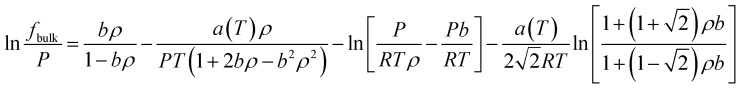
where *P* represents the pressure of gas in bulk.

The fugacity of CH_4_ taking into consideration the fluid–fluid interactions is expressed as,17
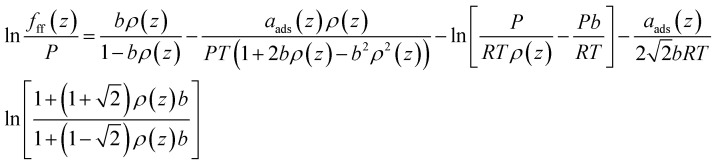
where the term *a*_ads_(*z*) correlates with the position in pores and the dimensionless pore size *L*/*σ*_ff_.^36^ Chen *et al.* (1997)^36^ proposed the equations for calculating the term *a*_ads_(*z*). *ρ*(*z*)represents the gas density in pores, which is a function of position in pores.

It has been found that the covolume parameter *b* in eqn (17) affects the adsorbed CH_4_ density near the pore surface.^30^ In order to consider the repulsive interactions of the adsorbed CH_4_, Fitzgerald (2005)^37^ modified this term to improve the predictive capacity of CH_4_ on pore surface. It is expressed as,^37^

18 *b*_ads_ = *b*(1 + *Λ*_*b*_)

where *b*_ads_ is the modified covolume; *Λ*_*b*_ represents the empirical correction, which is usually ranges from −0.4 to 0.0 for shale gas.^32^ In this work, this value is fixed at −0.20 for CH_4_. Eqn (17) is then rewritten as,19
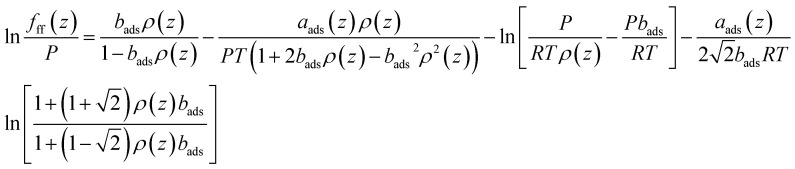


The density profile of CH_4_ in a pore is calculated by combining eqn (6) through (19). The excess adsorption is expressed as,20
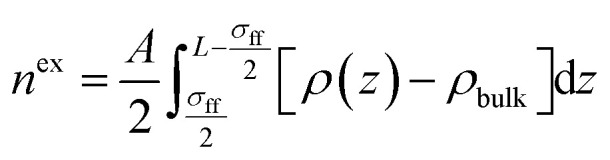
where *n*^ex^ represents the excess adsorption; *A* is the surface area. As for the integration of *σ*_ff_/2, the lower limit is the center of CH_4_ adsorbed on pore surface, while the upper limit *L* − (*σ*_*ff*_/2) represents the center of CH_4_ molecule adsorbed on pore surface.

The average density (*ρ*_ave_) of the adsorbed CH_4_ in nanopores is calculated by,21
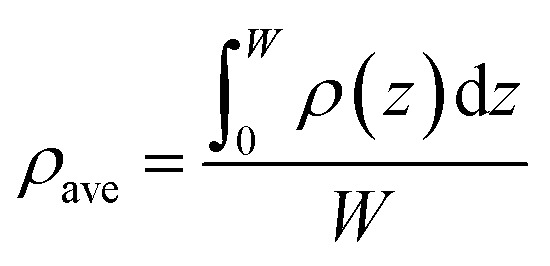
where *W* is the width of the adsorbed phase of CH_4_.

## 3. Results and discussion

In this subsection, characterization results of the shale samples are first presented. Then, we show the absolute CH_4_ adsorption calculated from the modified Langmuir adsorption model. SLD model is then employed to obtain the adsorbed CH_4_ density in pores. Using the calculated adsorbed CH_4_ density, the measured excess adsorption is then corrected to obtain the absolute adsorption. Finally, we evaluate the performance of the modified Langmuir adsorption model by comparing with SLD model.

### 3.1 Shale sample characterization

**Fig. 1:**
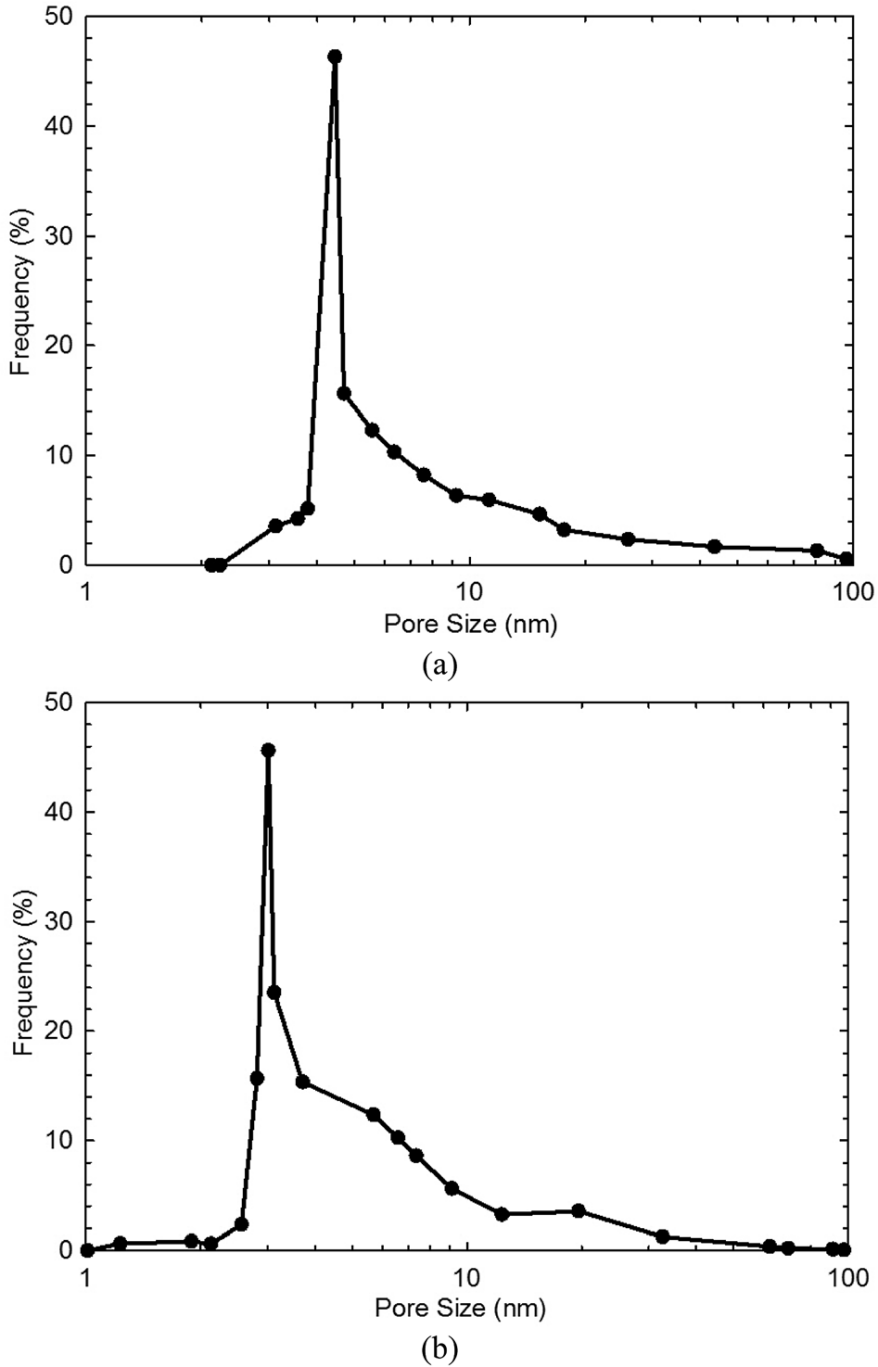
The measured pore size distribution of (a) shale sample-1, and (b) shale sample-2.

**Fig. 2:**
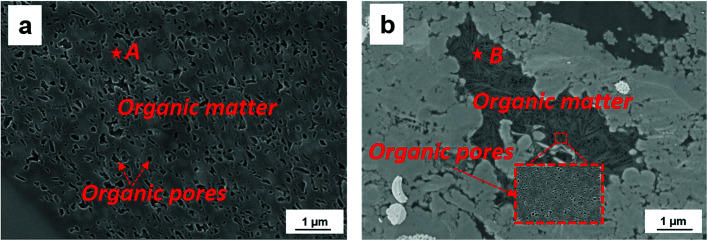
The SEM digital images of (a) shale sample-1, (b) shale sample-2.

Table 1 shows the measured TOC content and specific surface area of both shale samples. We find that the TOC content in shale sample-1 is higher than that in shale sample-2. However, the specific surface area of shale sample-1 is lower than that of shale sample-2. High TOC content indicates high content of kerogen in shale, which contributes to the specific surface area of shale samples. However, for given shale sample, the specific surface area also correlates with the clay content, heterogeneity, and pore size distribution *etc*. Fig. 1 shows the measured pore size distribution of both shale samples. Pores in both shale samples are generally in nano-scale locating in the range of 1–100 nm. The dominant pore sizes for the two shale samples are 4.35 nm and 3.12 nm, respectively. Fig. 2 presents the scanned SEM digital images for the shale samples. The X-ray spectroscopy analysis is conducted on the locations of A and B on both shale samples. We observe a high content of carbon element residing in the two points, indicting kerogen. As shown in Fig. 2, we can also observe a bunch of pores present in kerogen, which is recognized as a unique characteristic of kerogen in shale.

**Table 1:** The measured TOC content and specific surface area of both shale samples

Sample ID	TOC content (wt%)	Specific surface area (m^2^ g^−1^)
1	2.38	21.365
2	1.65	29.569

### 3.2 Absolute CH_4_ adsorption from the modified Langmuir adsorption model

**Fig. 3:**
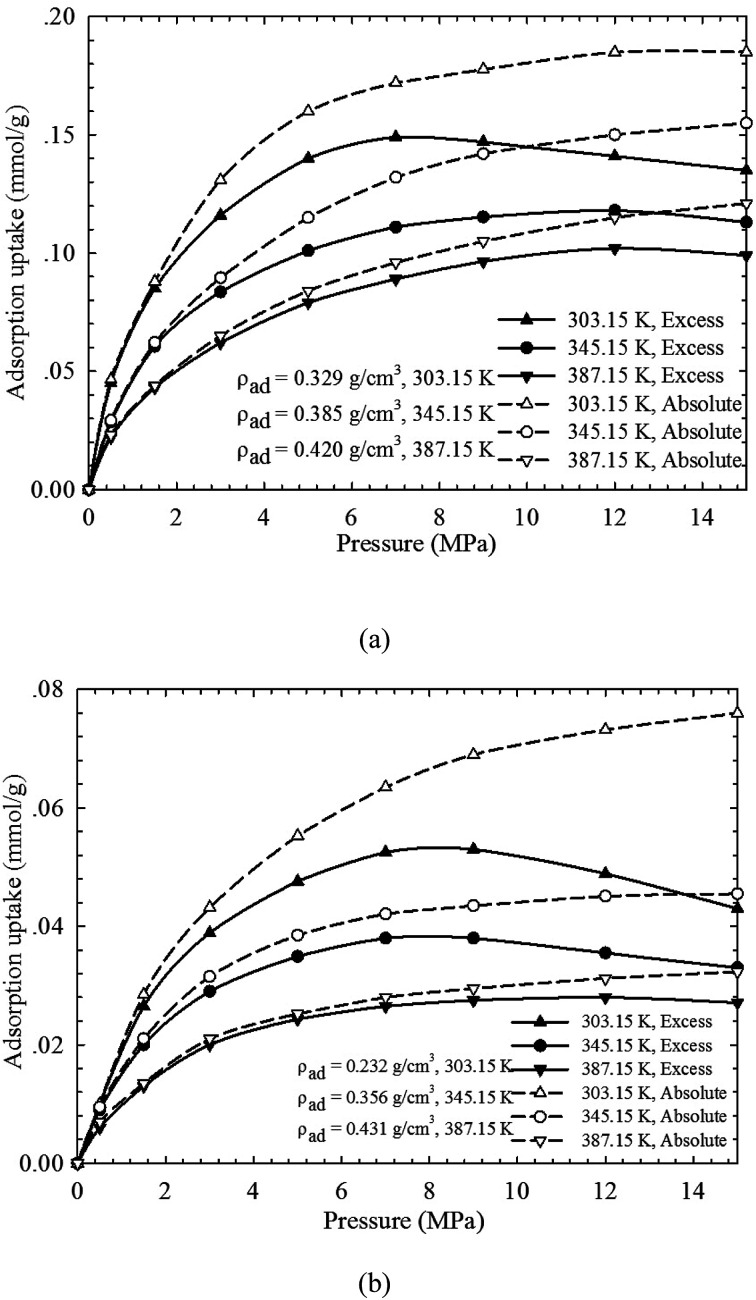
The measured excess adsorption and the calculated absolute CH_4_ adsorption on (a) shale sample-1, and (b) shale sample-2 from the modified Langmuir adsorption model.

Fig. 3 presents the measured excess CH_4_ adsorption on both shale samples at pressures up to 15.0 MPa and temperatures of 303.15–387.15 K. The modified Langmuir adsorption model is employed to fit the excess adsorption by adjusting the adsorbed CH_4_ density (*ρ*_ad_). We can observe that a perfect matching has been achieved between the measured results and the predicted values from the modified Langmuir adsorption model. At 303.15 K, the excess adsorption of CH_4_ is enhanced as pressure increases. The excess adsorption reaches the maximum at around 8.0 MPa on the two shale samples. However, as pressure further increases, the measured excess adsorption decreases. Tian *et al.* (2017)^2^ attributed this behavior to the much higher CH_4_ density at the center of organic pores at higher pressure conditions. The excess adsorption is then corrected to absolute adsorption using eqn (5) from the modified Langmuir adsorption model, as shown in Fig. 3. The absolute adsorption is clearly affected by the system temperature and pressure; specifically, it decreases with increasing temperature but increases as pressure increases. Moreover, compared to the excess adsorption, the absolute adsorption is higher, especially at higher pressure conditions, which agrees well with the previous findings.^2,12^ It suggests the amount of adsorbed CH_4_ on the organic shale is underestimated by the excess adsorption. In addition, the absolute CH_4_ adsorption varies for different shale samples at the same testing pressure/temperature conditions. Besides of system pressure and temperature, CH_4_ adsorption is expected to be also influenced by mineral contents, heterogeneity, specific surface area, and total organic carbon content *etc.*

### 3.3 Adsorbed CH_4_ density in nanopores

**Fig. 4:**
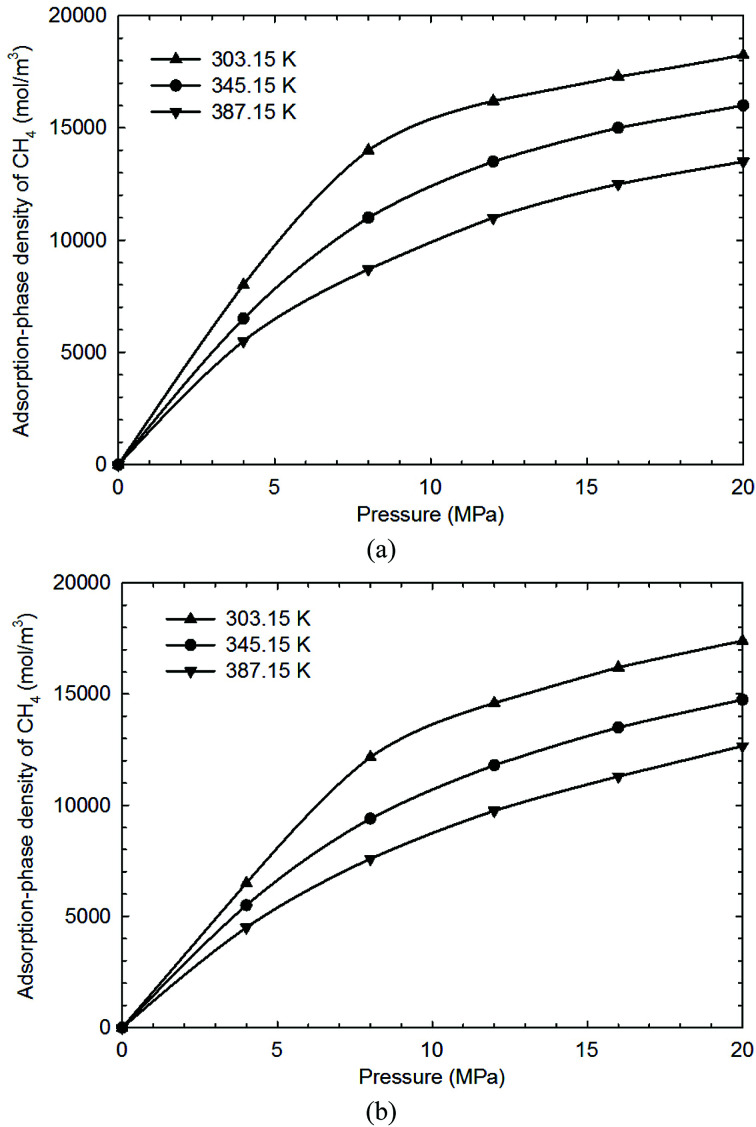
The adsorbed CH_4_ density in the carbon-slit pores of (a) 4.35 nm, and (b) 3.12 nm at different temperature and pressure conditions.

Using SLD model, we investigate the CH_4_ distribution in the 4.35 nm and 3.12 nm pores. Note that the most probable pore sizes of shale samples-1 and -2 are 4.35 nm and 3.12 nm, respectively. Based on the previous studies, it has been found that CH_4_ is single-layered adsorption in organic pores.^2,12^ As is known that the molecular diameter of CH_4_ is about 0.37 nm, previous works generally used 0.37 nm as the phase width of the adsorbed CH_4_. In our work, we also take 0.37 nm as the phase width of the adsorbed CH_4_ in nanopores. The average density of the adsorbed phase is calculated with 
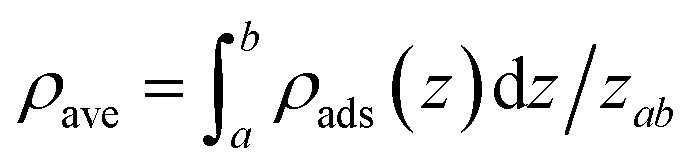
 (where *ρ*_ave_ represents the averaged adsorbed phase density of CH_4_; *ρ*_ads_ represents the *in situ* density in the adsorbed phase of CH_4_; and *z*_*ab*_ represents the phase width of the adsorbed CH_4_). Fig. 4 shows the calculated density of the adsorbed CH_4_ in the 4.35 nm and 3.12 nm pores at the experimental temperature/pressure conditions. We observe the adsorbed CH_4_ density is related with the experimental temperature and pressure. Specifically, the adsorbed CH_4_ density increases with increasing pressure but decreases as temperature increases. We observe that such density varies in the two different pores. Therefore, we may expect that the density of the adsorbed CH_4_ in is affected by temperature, pressure, and pore size. The previous works that employed constant values to represent the density of adsorbed CH_4_ is not physically reasonable.^13–17^

### 3.4 Absolute adsorption isotherms of CH_4_ from SLD model

**Fig. 5:**
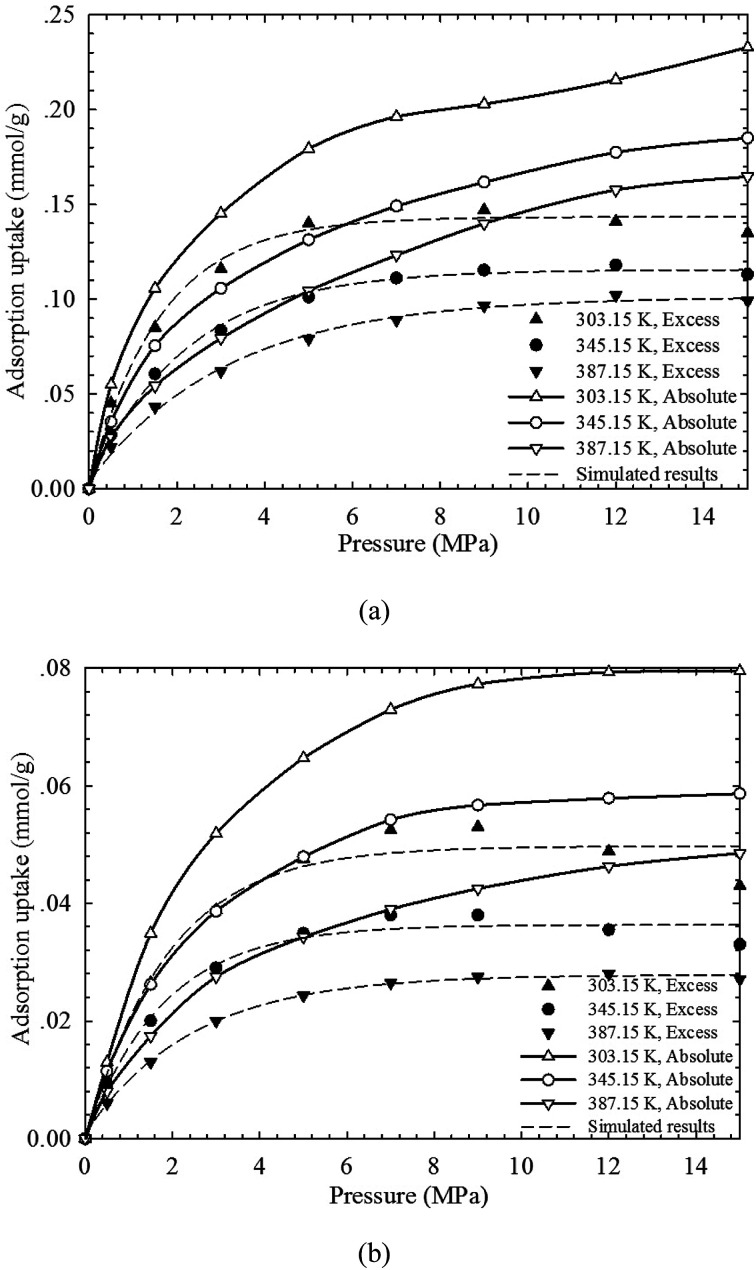
The measured excess adsorption and the calculated absolute CH_4_ adsorption on (a) shale sample-1, and (b) shale sample-2 from the SLD model.

In this work, two key parameters, *i.e.*, fluid-pore surface interaction energy (*ε*_fs_/*k*) and covolume correction parameter (*A*_*b*_), are adjusted in the SLD model. These parameters are obtained by adjusting these parameters to fit the measured excess adsorption. Table 2 shows the adjusted parameters in the SLD model for both shale samples. We observe that the covolume correction parameter is in the range of −0.3–0.3, which has a good agreement with the previous studies.^34,38,39^Fig. 5 shows the measured excess adsorption and the calculated absolute CH_4_ adsorption on both shale samples from the SLD model. We find that the SLD model can properly represent the excess CH_4_ adsorption. Moreover, the converted absolute adsorption is also greater than the measured excess, especially at high pressure conditions, which is similar to the observation from the modified Langmuir adsorption model.

**Table 2:** The key parameters used in the SLD model for the two shale samples

Core sample	*L* (nm)	*ε*_fs_/*k* (K)	*A*_*b*_	*A* (m^2^ g^−1^)
#1	4.35	78.6	0.039	21.365
	4.35	76.3	0.065	21.365
	4.35	72.1	0.046	21.365
#2	3.12	72.6	0.126	29.569
	3.12	70.9	0.139	29.569
	3.12	68.5	0.116	29.569

### 3.5 Evaluation of the modified Langmuir adsorption model

**Fig. 6:**
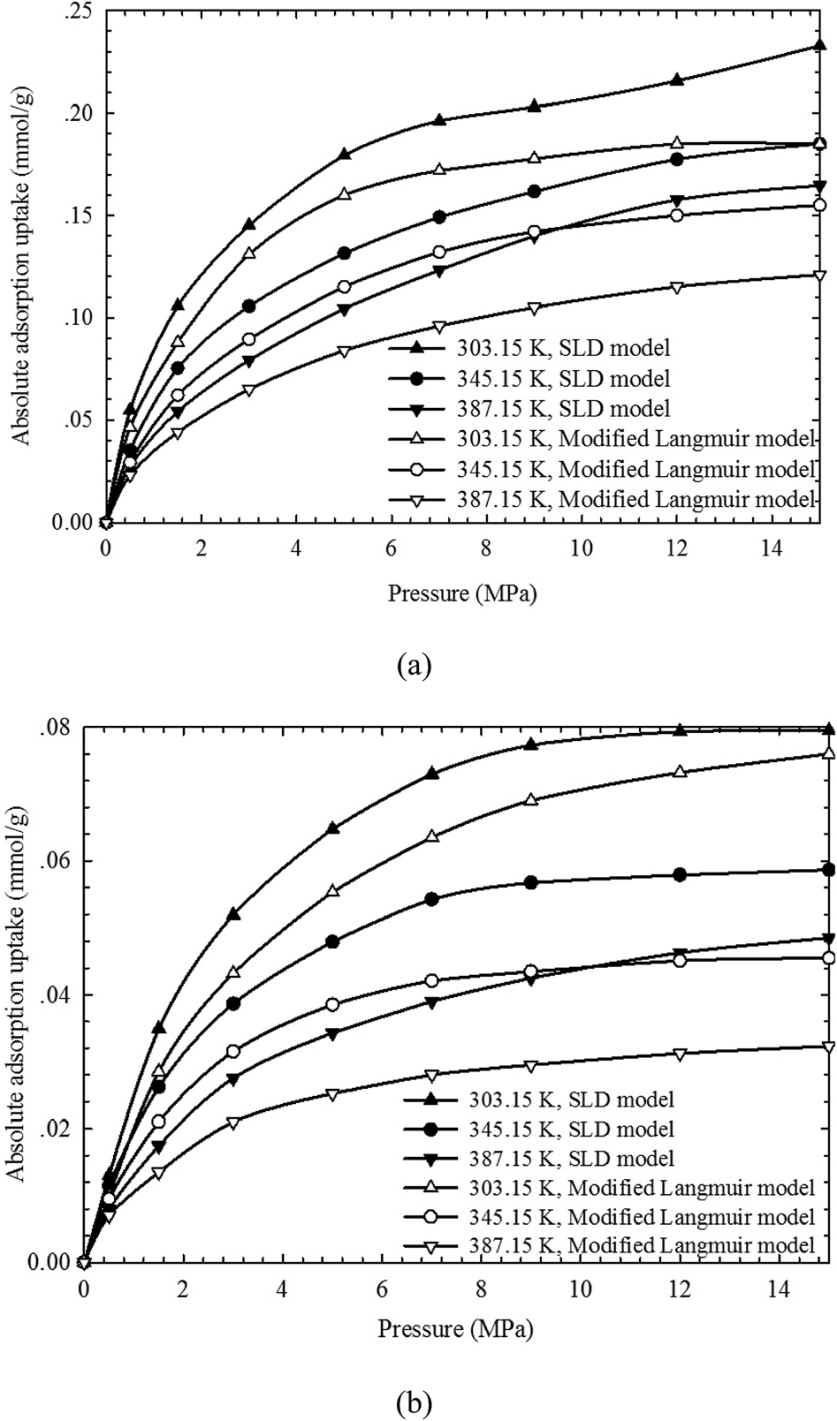
Comparisons of absolute adsorption isotherms between the SLD model and the modified Langmuir adsorption model.

It has been proved that SLD model can reasonably capture the adsorbed CH_4_ density and can thus accurately describe the absolute adsorption isotherms. In Fig. 6, the absolute adsorption isotherms calculated from SLD model are compared with those obtained from the modified Langmuir adsorption model. The performance of the modified Langmuir adsorption model is then evaluated in describing the absolute adsorption. We observe that the absolute adsorption obtained from SLD model are always higher than those obtained from the modified Langmuir adsorption model. The modified Langmuir adsorption model describes the absolute adsorption isotherm with constant density values representing the adsorbed CH_4_ density at a given temperature (see Fig. 3). However, based on the results calculated from SLD model, the density of adsorbed CH_4_ is related with the temperature, pressure, and pore size. Thereby, the widely used modified Langmuir adsorption model underestimates the actual adsorption and is not reasonable in obtaining the absolute CH_4_ adsorption on organic shale samples.

## 4. Conclusions

In this paper, the excess CH_4_ adsorption is measured on two shale core samples. We then use the modified Langmuir adsorption model and SLD model to fit the excess adsorption and then describe the absolute CH_4_ adsorption on the shale core samples. SLD model considers the fluid/pore surface interactions, which can thereby capture the density of adsorbed CH_4_ in nanopores. This study evaluates the performance of the modified Langmuir adsorption model in describing absolute adsorption of CH_4_ on organic carbon surface, and more importantly, it raises a more efficient approach (*i.e.*, SLD theory) than the sophisticated molecular simulation tools in determining the absolute adsorption. The detailed conclusions can be summarized as follows:

• Based on the simulation results from SLD model, the density of adsorbed CH_4_ is affected by temperature, pressure, and pore size. It highlights the importance for accurately determining the adsorbed CH_4_ density in obtaining the absolute CH_4_ absorption;

• It is found that the corrected absolute adsorption is greater than the excess CH_4_ adsorption on shale, especially at high pressures. It indicates that the measured excess CH_4_ adsorption shows underestimation of the amount of adsorbed CH_4_ on shale;

• Compared to the SLD model, the absolute adsorption obtained from the modified Langmuir adsorption model is always smaller than that obtained from the SLD model. It suggests that the absolute adsorption obtained from the modified Langmuir adsorption model underestimates the actual adsorbed CH_4_.

This study may inspire us new tools in determining the absolute adsorption uptake of CH_4_ on shale samples, which is practical in estimating the shale gas storage in shale gas reservoirs. The SLD model is more efficient in calculating the adsorbed CH_4_ density on shale than the molecular simulation methods. However, besides CH_4_, some heavier hydrocarbon components may also appear in shale fluids. Therefore, in the future works, the excess adsorption is suggested to be measured for the heavier hydrocarbon species and the SLD model recommended to calculate the adsorbed density for the heavier hydrocarbons on shale samples.

## Conflicts of interest

There are no conflicts to declare.

## Supplementary Material

## References

[cit1] Liu Y., Jin Z., Li H. (2018). Soc. Pet. Eng. J..

[cit2] Tian Y., Yan C., Jin Z. (2017). Sci. Rep..

[cit3] Clarkson C. R. and Haghshenas B., Texas, USA, 2013, vol. 10–12, SPE-164532-MS

[cit4] Wu Y., Fan T., Jiang S. (2015). et al.. Energy Fuels.

[cit5] Yan B., Wang Y., Killough J. E. (2016). Comput. Geosci..

[cit6] Lu X. C., Li F. C., Watson A. T. (1995). Fuel.

[cit7] Mertens F. O. (2009). Surf. Sci..

[cit8] Heller R., Zoback M. (2014). Journal of Unconventional Oil and Gas Resources.

[cit9] Gasparik M., Bertier P., Gensterblum Y. (2014). et al.. Int. J. Coal Geol..

[cit10] Myers A. L., Monson P. A. (2014). Adsorption.

[cit11] Zhou S., Wang H., Xue H. (2016). et al.. Nat. Gas Ind..

[cit12] Liu Y., Li H., Tian Y. (2018). et al.. Fuel.

[cit13] Lewis W. K., Gilliland E. R., Chertow B. (1950). et al.. Ind. Eng. Chem..

[cit14] Grant R. J., Manes M. (1964). Ind. Eng. Chem. Fundam..

[cit15] Menon P. G. (1968). J. Phys. Chem..

[cit16] Tsai M. C., Chen W. N., Cen P. L. (1985). et al.. Carbon.

[cit17] Wang Y., Zhu Y., Liu S. (2016). et al.. Fuel.

[cit18] Jin Z., Firoozabadi A. (2013). Fluid Phase Equilib..

[cit19] Ambrose R. J., Hartman R. C., Diaz-Campos M. (2012). et al.. Soc. Pet. Eng. J..

[cit20] Gensterblum Y., Hemert P., Billemont P. (2009). et al.. Carbon.

[cit21] Gensterblum Y., van Hemert P., Billemont P. (2010). et al.. Int. J. Coal Geol..

[cit22] Xiong F., Wang X., Amooie M. A. (2017). et al.. Fuel.

[cit23] Liu Y., Wang C. (2019). SPE J..

[cit24] Wang Y., Tsotsis T. T., Jessen K. (2015). Ind. Eng. Chem. Res..

[cit25] Hall F. E. , Zhou C. H., Gasem K. A. M., Robinson R. L. and Dan Y., SPE Eastern Regional Meeting, vol. 8–10, Charleston, West Virginia, 1994

[cit26] Gasparik M., Ghanizadeh A., Bertier P. (2012). et al.. Energy Fuels.

[cit27] Rexer T. F. T., Benham M. J., Aplin A. C. (2013). et al.. Energy Fuels.

[cit28] Yu L. J., Fan M., Chen H. Y., Zhang W. W., Xu E. (2015). Acta Pet. Sin..

[cit29] Krooss B. M., van Bergen F., Gensterblum Y., Siemons N., Pagnier H., David P. (2002). Int. J. Coal Geol..

[cit30] Gensterblum Y., Merkel A., Busch A., Krooss B. M. (2013). Int. J. Coal Geol..

[cit31] Rangarajan B., Lira C. T., Subramanian R. (1995). AIChE J..

[cit32] Mohammad S. A., Chen J. S., Robinson Jr R. L. (2009). et al.. Energy Fuels.

[cit33] Lee L. L. , Butterworths: Stoneham, MA, 1988

[cit34] Mohammad S. A., Arumugam A., Robinson Jr R. L. (2011). et al.. Energy Fuels.

[cit35] Gasem K. A. M., Gao W., Pan Z. (2001). et al.. Fluid Phase Equilib..

[cit36] Chen J. H., Wong D. S. H., Tan C. S. (1997). et al.. Ind. Eng. Chem. Res..

[cit37] Fitzgerald J. E. , PhD Dissertation, Oklahoma State University, Stillwater, OK, 2005

[cit38] Mohammad S., Chen J. S., Fitzgerald J. E. (2008). et al.. Energy Fuels.

[cit39] Pang Y., Mohamed Y. S., Sheng J. (2018). SPE Reservoir Eval. Eng..

